# Boi‐Ogi‐To, a Traditional Japanese Kampo Medicine, Promotes Cellular Excretion of Chloride and Water by Activating Volume‐Sensitive Outwardly Rectifying Anion Channels

**DOI:** 10.1096/fj.202403278R

**Published:** 2025-05-08

**Authors:** Kaori Sato‐Numata, Taro Suzuki, Haruna Saito, Shotaro Kato, Ayako Sakai, Shuntaro Mori, Hajime Nakae, Hitoshi Hasegawa, Yasunobu Okada, Tomohiro Numata

**Affiliations:** ^1^ Department of Integrative Physiology, Graduate School of Medicine Akita University Akita Japan; ^2^ School of Medicine Akita University Akita Japan; ^3^ Department of Pharmacy Akita University Hospital Akita Japan; ^4^ Department of Emergency and Critical Care Medicine Akita University Graduate School of Medicine Akita Japan; ^5^ Department of Medical Education Akita University Graduate School of Medicine Akita Japan; ^6^ National Institute for Physiological Sciences Okazaki Aichi Japan; ^7^ Department of Physiology, School of Medicine Aichi Medical University Nagakute Aichi Japan

**Keywords:** Boi‐Ogi‐to (BOT), cell volume regulation, edema, herbal medicine, translational science, VSOR

## Abstract

The Japanese Kampo medicine Boi‐ogi‐to (BOT) is known as an effective therapeutic agent for edema and nephrosis by promoting the excretion of excess body fluids. Despite its empirical effectiveness, scientific evidence supporting its effectiveness remains limited. In this study, we conducted a retrospective study of the effects of BOT administration on the blood test values of patients before and after taking the drug to attempt translational research between basic science and daily clinical practice by focusing on the molecular mechanism of action of BOT in vitro. We found that blood sodium and chloride levels are higher after taking BOT by analyzing the clinical test values before and after taking the drug from 28 patients attending Akita University Hospital. In this light, we measured the cell volume of human embryonic kidney HEK293T cells in vitro in order to investigate the possibility that BOT induces Cl^−^ excretion and cell volume reduction. BOT showed concentration‐dependent cell volume reduction with an EC_50_ of 686 μg/mL. The volume reduction effect was suppressed by the Cl^−^ channel inhibitors DIDS and DCPIB. Furthermore, patch‐clamp studies showed that BOT‐activated Cl^−^ currents exhibit outward rectification and time‐dependent inactivation upon depolarization. These biophysical properties of BOT‐induced Cl^−^ currents correspond to those of volume‐sensitive outward rectifier (VSOR) anion channels. The Cl^−^ currents activated by the administration of BOT were inhibited by applying DIDS, DCPIB, and siRNA targeting the gene of LRRC8A, a core component of the VSOR channel, as well as in LRRC8‐deficient cells. Additionally, BOT‐induced Cl^−^ currents were restored by coexpression of LRRC8A/C in LRRC8‐deficient cells. Also, BOT was found to translocate LRRC8A proteins to the plasma membrane. These results demonstrated that BOT activates LRRC8‐containing VSOR channels by delivering LRRC8A to the plasma membrane and induces Cl^−^ release, thereby promoting water excretion.

## Graphical abstract

Boi‐ogi‐to (BOT) facilitates excretion of Cl^−^ and water by activating VSOR anion channels. This study shows that BOT increases serum Na^+^ and Cl^−^ levels in laboratory values and induces Cl^−^ currents in human kidney epithelial HEK293T cells. Patch‐clamp studies confirmed that BOT activates LRRC8‐containing VSOR channels by translocating LRRC8A to the plasma membrane, thereby promoting cellular volume decreases and water excretion.

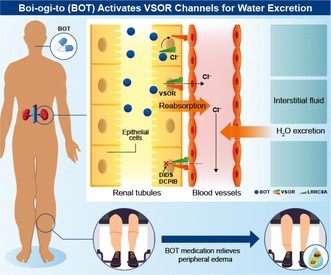

## 1 Introduction

Edema is characterized by the accumulation of excessive fluid in the interstitial spaces, a condition commonly observed in elderly individuals, especially women [1]. Left untreated, edema can lead to symptoms such as swelling‐induced pain, itching, and reduced blood flow, ultimately affecting various aspects of health. Therefore, addressing edema is an important clinical concern.

Boi‐ogi‐to (BOT) is a traditional Japanese Kampo medicine known for its anti‐edema, anti‐inflammatory, diuretic, and sweat‐reducing effects. It is used to treat morbid conditions such as arthritis, nephrotic syndrome, and obesity, particularly in patients experiencing symptoms like fatigue, poor complexion, and peripheral edema.

BOT is typically administered to adults at a dose of 1.875 g per dose, taken twice daily. Each dose contains a standardized extract composed of six herbal ingredients in the following proportions: 1.25 g of Boi (*Sinomenium acutum*), 1.25 g of Ogi (*Astragalus membranaceus*), 0.75 g of Taiso (*Jujube*), 0.375 g of Kanzo (*Glycyrrhiza*), 0.25 g of Shokyo (*Ginger*), and 0.75 g of Sojyutsu (*Atractylodes*). Some of these components are known to modulate the activity of ion channels, suggesting their involvement in the mechanism for the therapeutic effects of BOT. Sinomenine, an alkaloid extracted from Boi, has been demonstrated to exert anti‐inflammatory effects and is known to regulate some cation channels. For example, studies in ischemic brain injury models have shown that sinomenine protects neurons by inhibiting acid‐sensing ion channels (ASIC1a) and L‐type Ca^2+^ channels [2]. Sinomenine also reduces pain by inhibiting Nav channels in dorsal root ganglion (DRG) neurons [3]. Kanzo contains licochalcone A, a natural chalcone with anti‐inflammatory, antioxidant, and antitumor effects. Licochalcone A has been shown to inhibit several cation channels, including Orai1, Kv1.3, and KCa3.1, in a concentration‐dependent manner [4]. Another compound of Kanzo, isoliquiritigenin, exerts analgesic effects by inhibiting Nav channels [5]. Shokyo contains 6‐gingerol, which activates Transient Receptor Potential (TRP) C5 and TRPA1 channels [6] but inhibits the activity of hERG K^+^ channel [7], L‐type Ca^2+^ channel [8], and Nav channel [9]. Sojyutsu contains atractylodin, which has been shown to exert analgesic effects by inducing long‐term activation of TRPA1 channels [10]. These findings suggest that the bioactive compounds found in BOT may serve as potential therapeutic agents by regulating physiological processes through ion channel modulation.

Recent studies using rat models of knee arthritis have suggested that BOT may reduce joint effusion by affecting some channels, particularly aquaporins (AQPs), which are essential for water transport [11]. Since net water transport can be driven by flows of osmolytes, the potential importance of not only cation channels but also anion channels must be highlighted in water transport regulating fluid balance and edema. However, the specific anion channel‐mediated mechanisms behind these effects have remained elusive.

In this study, our goal is to explore the effects of BOT on clinical laboratory parameters and to identify the specific anion channels involved in BOT‐induced fluid excretion using human embryonic kidney HEK293T cells.

## 2 Methods

### 2.1 Reagents

Dimethyl sulfoxide (DMSO) was purchased from Wako Pure Chemical Industries Ltd. (Osaka, Japan). 4,4′‐Diisothiocyanato‐2,2′‐stilbenedisulfonic acid disodium salt (DIDS) and 4‐[(2‐butyl‐6,7‐dichloro‐2‐cyclopentyl‐2,3‐dihydro‐1‐oxo‐1h‐inden‐5‐yl) oxy] butanoic acid (DCPIB) were obtained from Sigma‐Aldrich (St. Louis, MO, USA) and Tocris (Bristol, UK), respectively. Boi‐ogi‐to (TJ‐20) was obtained from TSUMURA & CO (Tokyo, Japan). Boi‐ogi‐to powder was dissolved in DMSO at a concentration of 800 mg/mL for same‐day use. All other chemical reagents were purchased from commercial suppliers.

### 2.2 Animals

The animal experiment protocol was approved by the Akita University Animal Ethics Committee (Akita, Japan; Ethics Review Numbers: a‐1‐0412). Male C57BL/6 mice, aged 8–12 weeks, were housed in a controlled environment at 22°C–25°C, under a 12‐h light–dark cycle, with free access to water and a commercial diet.

### 2.3 Cell Culture, Downregulation of LRRC8A Expression, and Overexpression of LRRC8A/C

Human Embryonic Kidney (HEK) 293T cells, HEK293 cells with disruption of all five LRRC8 genes (HEK LRRC8^−/−^) cells (kindly provided by Dr. Thomas J Jentsch [12, 13]), human colon epithelial Caco‐2 cells, and human cervix epithelial HeLa cells were used in this study. HEK293T cells were cultured in Dulbecco's Modified Eagle's Medium (DMEM), supplemented with 10% fetal bovine serum, 30 units/mL penicillin, and 30 μg/mL streptomycin. For HeLa cells, Minimum Essential Medium (MEM) was used instead of DMEM. All cells were maintained at 37°C in a humidified atmosphere of 95% air and 5% CO_2_. Renal tubular cells were isolated from the renal cortex of male C57BL/6 mice. Briefly, the renal cortex was finely minced using scissors, followed by collagenase treatment to dissociate the tissue. The resulting suspension was subjected to filtration through a 100 μm mesh filter. The filtrate was then further processed using a 70 μm mesh filter to isolate the specific cell population retained on the filter. After filtration, the enzyme was removed by washing the cells with phosphate‐buffered saline (PBS). The cell suspension was then resuspended in DMEM, gently mixed by pipetting, and plated onto culture dishes. The cells in primary culture were used for experiments within 48 h. To reduce the expression of human LRRC8A, siRNA‐mediated knockdown was performed in HEK293T cells at 70%–80% confluency. For siRNA transfection, the Lipofectamine RNAiMAX Transfection Reagent (Invitrogen, Carlsbad, CA, USA) was employed according to the manufacturer's protocols. The LRRC8A‐specific siRNA [14] conjugated with HiLyte 488 was designed and synthesized by NIPPON GENE Co. (Toyama, Japan). A negative control siRNA conjugated with Alexa Fluor 488 (Allstars; Qiagen, Hilden, Germany) was utilized in control transfections. Cells were used in experiments 34–48 h after siRNA transfection. To observe the localization of LRRC8A protein expression, the cells after 24‐h plating were transfected with either EGFP‐F vector (Clontech Laboratories Inc., CA, USA) with or without human LRRC8A‐mCherry vector [15], using Lipofectamine 2000 (Invitrogen) as the transfection reagent, according to the manufacturer's protocol. To investigate the effect of BOT on the VSOR channel formed with LRRC8A *plus* LRRC8C, HEK‐LRRC8^−/−^ cells were cultured for 24 h and then transfected using Lipofectamine 2000 (Invitrogen) with pIRES2‐AcGFP1‐human LRRC8A and pIRES2‐DsRed‐human LRRC8C vectors. The LRRC8C plasmid (Sino Biological Inc., Beijing, China) was cloned into the pIRES2‐DsRed vector. Both pIRES2‐AcGFP1 and pIRES2‐DsRed were obtained from Takara Bio (Shiga, Japan).

### 2.4 Confocal Microscopy

Cells transfected with plasmids were fixed with 4% paraformaldehyde and then permeabilized with 0.1% Triton X‐100. The cells were incubated with anti‐GFP (1:1000 dilution, 50430‐2‐AP; Proteintech, IL, USA) or anti‐mCherry (1:1000 dilution, GTX630189; GeneTex Inc., CA, USA) primary antibodies, followed by incubation with Alexa Fluor 488‐conjugated anti‐rabbit (1:4000 dilution, A‐1100; Thermo Fisher Scientific, Waltham, MA, USA) or Alexa Fluor 647‐conjugated anti‐mouse (1:4000 dilution, A‐21236; Thermo Fisher Scientific) secondary antibodies. After washing with PBS, the cells were mounted on glass slides using ProLong Diamond Antifade Mountant with DAPI (Thermo Fisher Scientific). Fluorescence images were acquired with a confocal laser‐scanning microscope (Zeiss LSM970; Carl Zeiss Microscopy GmbH, Jena, Germany), which equips a × 40 oil objective lens. Alexa Fluor 488, Alexa Fluor 647, or DAPI signals were acquired, and line analyses were performed with ZEN software (Carl Zeiss).

### 2.5 Mean Cell Volume and Cell Size Measurements

The mean cell volume in HEK239T, HeLa, and Caco‐2 cells was measured at room temperature using a Coulter‐type cell size analyzer (CDA‐500; Sysmex, Kobe, Japan). Isotonic “Tyrode solution” (300 mosmol/kg‐H_2_O adjusted by D‐mannitol) contained (in mM): 140 NaCl, 5 KCl, 1 MgCl_2_, 2 CaCl_2_, 10 D‐glucose, and 10 HEPES (pH 7.4 adjusted by NaOH). Relative cell volume was calculated using the following equation: Relative cell volume = *V*_*A*_/*V*_*Ctl*_, where *V*_*Ctl*_ and *V*_*A*_ represent the mean cell volumes before and after administration of BOT, respectively, with or without DIDS or DCPIB. Renal tubular cells attached to coverslips were preincubated in isotonic solution (300 mosmol/kg‐H_2_O) for 10 min, and the single‐cell size was measured at room temperature. Tyrode solution was used as the experimental medium. Cells were visualized using a microscope equipped with a low‐light observation camera (WRAYCAM‐VEX; WRAYMER Co. Ltd., Osaka, Japan), and images were recorded with the software (WRAYMER Co. Ltd.). The cell size of primary renal tubular cells was quantified by measuring the cross‐sectional area (CSA) of the cell body using ImageJ software [16].

### 2.6 Cell Counting Assay

HEK293T cells (1 × 10^5^ cells) were reseeded in 6‐cm dishes and cultured in Tyrode solution with or without BOT for 24 h. Thereafter, 1 μL of acridine orange/propidium iodide (AO/PI) solution obtained from a cell viability kit (Logos Biosystems Inc., Anyang, Republic of Korea) was added to 100 μL of each sample, following cell detachment by gentle pipetting. The samples were incubated at room temperature for 10 min, after which the stained cells were loaded onto counting slides. Images of the samples were captured using a Countess II‐FL automated cell counter (Thermo Fisher Scientific). AO‐positive cells were considered viable, while PI‐positive cells, relative to AO‐positive cells, were identified as dead cells. The cell proliferation at 24 h was evaluated by the percent of the number of AO‐positive cells at 24 h compared to that of the AO‐positive cells at 0 h.

### 2.7 Electrophysiology

HEK293T cells, siRNA‐transfected HEK293T cells, and HEK LRRC8^−/−^ cells were mechanically dispersed by pipetting and fixed onto glass coverslips placed in handmade chambers. Membrane currents of these cells were recorded at room temperature (22°C–27°C) by patch‐clamp whole‐cell recordings with an Axopatch 200B (Axon Instruments/Molecular Devices, Union City, CA, USA). Patch electrodes prepared from borosilicate glass capillaries had an input resistance of 3–5 MΩ when filled with solution. Current signals were filtered at 5 kHz with a four‐pole Bessel filter, digitized at 20 kHz, and recorded on a desktop computer. pCLAMP (version 10.5.1.0; Axon Instruments/Molecular Devices) software was used for command pulse control, and for data acquisition and analysis. Data were also analyzed using Origin (OriginLab Corp., Northampton, MA, USA) software. Series resistance was compensated for to minimize voltage errors (70%–80%). The external solution contained (in mM) 110 CsCl, 5 MgSO_4_, and 10 HEPES (pH 7.4 adjusted with CsOH, and osmolality adjusted to 330 mmol/kg with D‐mannitol). The pipette solution contained (in mM) 110 CsCl, 2 MgSO_4_, 1 EGTA, 10 HEPES, and 2 Na_2_ATP (pH 7.3 adjusted with CsOH, and osmolality adjusted to 300 mmol/kg with D‐mannitol). To modify the Cl^−^ concentration gradient, extracellular CsCl was replaced with an equivalent volume of Cs‐gluconate. To record the Cl^−^ currents under a physiological Cl^−^ gradient, the following solutions were employed. The external solution contained 120 mM CsCl, 5 mM MgSO_4_, and 10 mM HEPES, with the pH adjusted to 7.4 using CsOH and an osmolality of 330 mmol/kg, adjusted with D‐mannitol. The intracellular (pipette) solution contained 45 mM CsCl, 75 mM Cs‐gluconate, 2 mM MgSO_4_, 1 mM EGTA, 10 mM HEPES, and 2 mM Na_2_ATP with the pH adjusted to 7.3 using CsOH and the osmolality of 300 mmol/kg adjusted with D‐mannitol. Resting membrane potential measurements were performed using nystatin‐perforated whole‐cell recordings [17]. Briefly, the Na^+^‐based bath solution consisted of 140 mM NaCl, 5 mM KCl, 2 mM CaCl_2_, 1 mM MgCl_2_, 1 mM HEPES, and 10 mM D‐glucose with the pH adjusted to 7.4 using NaOH and the osmolality of 320 mOsmol/kg H_2_O, adjusted with D‐mannitol. The pipette solution for these recordings contained 55 mM K_2_SO_4_, 20 mM KCl, 5 mM MgCl_2_, 0.2 mM EGTA, and 5 mM HEPES, with the pH adjusted to 7.4 using KOH and an osmolality of 300 mOsmol/kg H_2_O, adjusted with D‐mannitol. An Ag‐AgCl pellet with a 3 M KCl‐agar bridge was used as the reference electrode.

### 2.8 RNA Isolation and RT‐PCR

Total cellular RNA was extracted from HEK293T cells by using NucleoSpin RNA Plus (Takara‐Bio, Shiga, Japan) according to the protocol supplied by the manufacturer. The concentration and purity of RNA were determined using a Nanodrop‐ND1000 (Thermo Fisher Scientific). Total RNA samples were reverse‐transcribed with ReverTra Ace qPCR RT Master Mix with gDNA Remover (Toyobo, Osaka, Japan), according to the manufacturer's protocols. Expression levels of LRRC8A in the cDNA from HEK293T cells were determined by PCR. As a positive control, we amplified the partial sequence of GAPDH. PCR was done using KOD‐Plus‐Neo (Toyobo) under the following conditions: Predenaturation at 94°C for 2.5 min, followed by 24 cycles of denaturation at 94°C for 15 s and annealing at 57°C for 30 s, and a final extension at 68°C for 45 s. The sequences of gene‐specific primers were previously reported [14].

### 2.9 Study Design for the Effects of Boi‐Ogi‐To (BOT) on Clinical Laboratory Data

A retrospective case study was conducted at Akita University Hospital from January 2012 to December 2022. The study included patients who initiated BOT therapy during this period and had available clinical laboratory data both before and after starting BOT treatment. BOT (TJ‐20, Tsumura Corporation, Tokyo, Japan) was prescribed at a daily dose of 2–3 sachets (2.5 g each) and administered orally in two or three divided doses. Additionally, this prescription was newly introduced to patients with no prior history of BOT use. Only laboratory data collected while the patients were actively taking BOT were included, and any data obtained after discontinuation of BOT were excluded. The follow‐up period for clinical laboratory values after BOT initiation was up to 1 year from the initial prescription. Patients without clinical laboratory data before or after BOT initiation were excluded from the study. All data were extracted from the medical records.

The values of each clinical laboratory parameter during the BOT treatment period were compared with the corresponding pre‐treatment values. Blood test values evaluated for all 28 patients included alanine aminotransferase (ALT), aspartate aminotransferase (AST), chloride, potassium, and sodium. Due to limited testing, albumin levels were evaluated in 22 patients, and systolic blood pressure (SBP) and diastolic blood pressure (DBP) were evaluated in 4 patients. Statistical analysis was performed using the Wilcoxon signed‐rank test, with a significance level of 5% (two‐sided). A normality test was also performed. EZR software [18] was used for all statistical analyses.

### 2.10 Institutional Review Board Statement

This study was performed in compliance with the tenets of the Declaration of Helsinki for experiments under the ethical approval issued by Akita University Institutional Review Board (No. 3113).

### 2.11 Statistical Evaluation

Experimental data are expressed as means ± SEM, with results derived from at least three independent experiments for each condition. Statistical analyses were performed using Student's *t* test, with a *p* value of < 0.05 considered significant. For the analysis presented in Figure 8B, a χ^2^ test was employed to evaluate the association between two categorical variables, with a value of *p* < 0.05 considered significant.

## 3 Results

### 3.1 BOT Increases Chloride and Sodium Concentrations in the Blood

Despite its empirical effectiveness against peripheral swelling and water retention, scientific evidence supporting the efficacy of the herbal medicine Boi‐ogi‐to (BOT) remains limited. Here, we examined the effects of BOT on clinical test values in patients treated at Akita University Hospital. The characteristics of the 28 patients included in this study are shown in Table 1. A total of 28 patients who met the inclusion criteria were analyzed before and after up to 1 year of BOT administration.

**TABLE 1:** Characteristics of patients subjected to BOT therapy.

*n* = 28	(%)
Gender	
Male	5 (17.9)
Female	23 (82.1)
Age	62.0 ± 17.9
Primaly desease	
Cancer	7 (25.0)
Collagen disease	4 (14.3)
Diabetes	2 (7.1)
Others	15 (53.6)
Concomitant drugs	
ARB	6 (21.4)
Candesartan Cilexetil	2 (7.1)
Irbesartan	1 (3.5)
Telmisartan	3 (10.7)
CCB	5 (17.9)
Amlodipine Besilate	3 (10.7)
Nifedipine	2 (7.1)
Diuretic	2 (7.1)
Furosemide	1 (3.5)
Indapamide	1 (3.5)
Baseline of clinical laboratory values	
ALT (IU/L)	28.9 ± 30.8
AST (IU/L)	27.0 ± 16.5
Albumin (g/dL)	4.1 ± 0.4
Serum creatinine (mg/dL)	0.7 ± 0.2
Chloride (mEq/L)	104.5 ± 2.4
Potassium (mEq/L)	4.3 ± 0.4
Sodium (mEq/L)	140.9 ± 1.9

*Note:* Continuous variables are expressed as mean ± standard deviation (SD), while categorical variables are shown as *n* (%).

Abbreviations: ACE, angiotensin‐converting enzyme; ARB, angiotensin‐receptor blocker; CCB, calcium channel blocker.

As shown in Table 2, the statistical analysis of laboratory values showed a small but statistically significant increase in serum sodium (Na) and chloride (Cl) concentrations by the fourth week after BOT treatment. When the more detailed time course was analyzed, these effects were again observed 3–5 weeks but were not detected in 1–2 and 6–8 weeks (Table 3). These results suggest that BOT exerts its therapeutic action in a relatively acute fashion. In fact, seven of the 28 patients exhibited laboratory findings indicative of reduced peripheral edema. Among them, four showed improvements within 3 weeks, two within 3 months, and one within 1 year. These findings are consistent with the onset of the edema‐improving effect of BOT beginning around the third week of administration to the Japanese patients subjected to chemoradiotherapy for recurrent vaginal squamous cell carcinoma, as published by Matsuoka et al. [19].

**TABLE 2:** Comparison of clinical laboratory values before and after administration of Boi‐ogi‐to (BOT).

Clinical laboratory values	Before	2–4 weeks	*p*
ALT (IU/L)	19.5 (15.0–29.3)	23.0 (16.8–26.8)	0.919
AST (IU/L)	23.0 (20.0–26.0)	20.5 (16.8–26.0)	0.476
Albumin (g/dL)	4.2 (3.9–4.4)	4.0 (3.8–4.2)	0.373
Chloride (mEq/L)	104.5 (103.0–105.3)	106.0 (105.0–106.3)	0.013*
Potassium (mEq/L)	4.3 (4.0–4.6)	4.0 (3.9–4.4)	0.106
Sodium (mEq/L)	141.0 (139.0–142.3)	142.0 (140.3–143.0)	0.012*
SBP (mmHg)	120.0 (118.5–124.0)	128.0 (124.0–129.0)	0.371
DBP (mmHg)	70.0 (68.5–71.5)	73.0 (71.5–76.5)	0.371

*Note:* Data are presented as median (interquartile range). Differences in laboratory values before and after BOT administration were analyzed using the Wilcoxon signed‐rank test. Statistically significant differences are denoted with *p < 0.05. Parameters measured include alanine aminotransferase (ALT), aspartate aminotransferase (AST), albumin, major electrolyte concentrations (chloride: Cl, potassium: K, sodium: Na), systolic blood pressure (SBP), and diastolic blood pressure (DBP).

**TABLE 3:** Time‐dependent changes in clinical laboratory values of electrolyte concentrations after administration of Boi‐ogi‐to (BOT).

Clinical laboratory values	Before	1–2 weeks	*p*	3–5 weeks	*p*	6–8 weeks	*p*
Chloride (mEq/L)	104.5 (103.0–105.3)	104.0 (103.0–106.0)	0.209	106.0 (105.3–107.8)	0.005*	104.0 (103.3–104.8)	1.000
Potassium (mEq/L)	4.3 (4.0–4.6)	4.0 (3.9–4.2)	0.062	4.1 (3.9–4.5)	0.271	4.1 (3.9–4.4)	0.892
Sodium (mEq/L)	141.0 (139.0–142.3)	141.0 (140.0–141.5)	0.763	142.0 (140.3–143.0)	0.009*	139.5 (138.3–140.0)	0.572

*Note:* Data are presented as median (interquartile range). Differences in laboratory values before and after BOT administration were analyzed using the Wilcoxon signed‐rank test. Statistically significant differences are denoted with *p < 0.05. Parameters measured include major electrolyte concentrations (chloride: Cl, potassium: K, sodium: Na).

This small but detectable increase in Na and Cl concentrations may represent a physiological response to BOT and may contribute to the observed therapeutic effect on reducing peripheral edema and water retention. These results suggest that BOT may selectively modulate specific ion concentrations in the blood, which could be an underlying mechanism of its therapeutic action.

### 3.2 BOT Induces Cell Volume Reduction via Cl^−^ Channel Activity

Our retrospective study indicated that BOT administration slightly but significantly increased plasma sodium and chloride concentration in 2–5 weeks by facilitating the absorption of chloride into the body or chloride release from the cells (Tables 2 and 3). Chloride efflux is known to be often associated with cell volume decrease driven by the cellular ion transport system [20, 21, 22], and this function may reduce edema by increasing plasma Cl concentration [23]. To further investigate the effects of BOT on cell volume, we measured cell volume changes using a Coulter counter in the HEK293T cell line.

**FIGURE 1:**
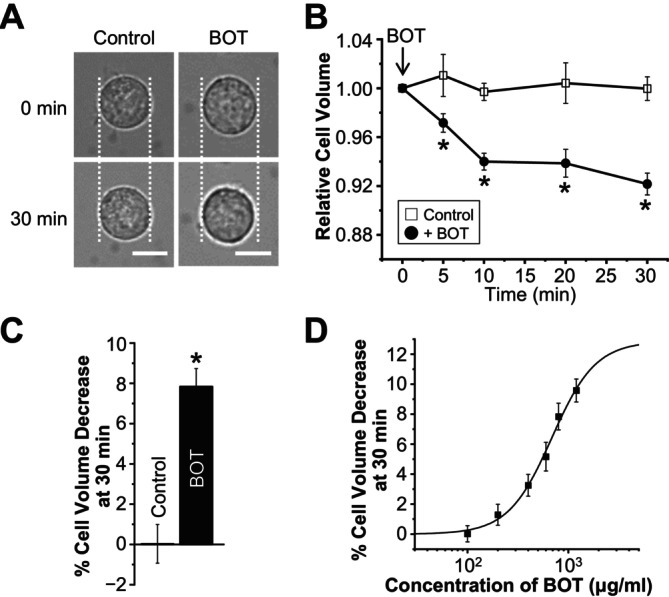
Boi‐ogi‐to (BOT) induces cell volume reduction in HEK293T cells. (A) Representative transmission microscopy images of HEK293T cells at 0 and 30 min in the absence (Control) and presence of BOT. Scale bar indicates 50 μm. (B) Time course of mean cell volume changes in HEK293T cells. BOT (800 μg/mL) was applied at 0 min, except in the control condition (*n* = 7–15). (C) Percentages of cell volume decrease at 30 min in the control and BOT‐treated cells compared to their initial cell volume (*n* = 7–15). (D) Concentration‐dependent cell volume decreases induced by 30‐min application of BOT with different concentrations. The data were fitted using a Hill curve, with an EC_50_ of 686.17 ± 121.4 μg/mL and a Hill coefficient of 2.0. (*n* = 5–15). *p < 0.05 compared to the Control.

**FIGURE 2:**
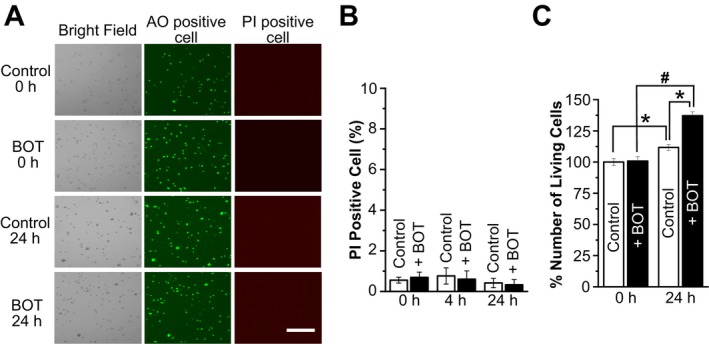
Boi‐ogi‐to (BOT) does not affect cell viability. (A) Representative bright‐field transmission microscopy images of AO‐ and PI‐stained HEK293T cells in the control and BOT‐treated (800 μg/mL) groups at 0 min and 24 h. Scale bar indicates 300 μm. (B) Percentage of PI‐positive (dead) cells relative to AO‐positive (alive) cells in the control and BOT‐treated (800 μg/mL) groups at 0, 4, and 24 h (*n* = 6–18). (C) Number of AO‐positive cells in the control and BOT‐treated (800 μg/mL) groups at 0 and 24 h (*n* = 12–19). *p < 0.05 compared to Control; ^#^ *p* < 0.05 compared to 0 h. The data at 0 h were calculated by normalizing individual control values to the average cell count of the control group at the 0‐h time point. Similarly, for the 24‐h data, the percentage of live cells was determined by normalizing each value to the average cell count at 0 h. The same normalization approach was applied to the BOT‐treated group.

As shown in Figure 1A–C, BOT treatment induced cell volume decreases in a time‐dependent manner and resulted in approximately a 7.8% cell volume decrease after 30 min, while no significant change in the cell volume was observed in the control group without BOT treatment. This cell volume decrease was found to be concentration‐dependent, with an EC_50_ of 686.2 ± 121.4 μg/mL (Figure 1D). Importantly, cell viability assays revealed that 24 h treatment with BOT did not induce cell death (Figure 2), indicating that the cell volume decrease observed was not due to cytotoxic effects.

**FIGURE 3:**
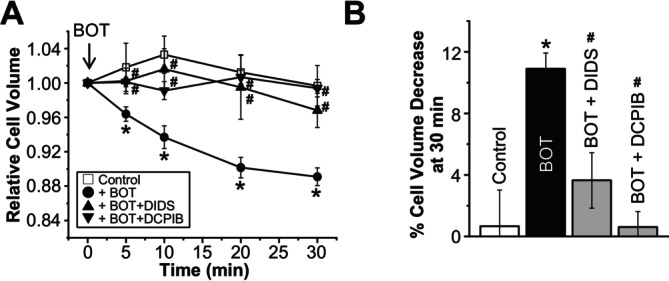
Cl^−^ channel inhibitors prevent cell volume reduction induced by Boi‐ogi‐to (BOT). (A) Time course of changes in the mean cell volume of HEK293T cells in the absence (Control) or presence of BOT (800 μg/mL) alone or together with 100 μM DIDS or 5 μM DCPIB. (*n* = 6–13) (B) Percentage of cell volume decrease measured at 30 min after BOT administration, calculated from the data in A. *p < 0.05 compared to Control; ^#^ *p* < 0.05 compared to BOT alone.

Given that Cl^−^ efflux is a well‐established mechanism associated with cell volume decrease [20, 21], we next explored the involvement of Cl^−^ channels in BOT‐induced cell volume decrease. To this end, we applied DIDS, a broad‐spectrum Cl^−^ channel inhibitor, and DCPIB, a relatively selective inhibitor of swelling‐activated Cl^−^ channel [24] which is called the volume‐sensitive outwardly rectifying (VSOR) anion channel [25] or the volume‐regulated anion channel (VRAC) [26]. Both inhibitors significantly suppressed BOT‐induced cell volume decrease (Figure 3), indicating that the Cl^−^ efflux through VSOR channels plays a key role in this process. Similar DIDS/DCPIB‐sensitive cell size reduction was observed after exposure to BOT in mouse renal tubular cells in primary culture and human colonic Caco‐2 cells but not in human cervical cancer HeLa cells, as presented in Supplementary Figure 1.

These findings suggest that BOT‐induced cell volume reduction in HEK293T and Caco‐2 cells as well as in primary renal tubular cells is mediated mainly by Cl^−^ efflux and accompanying water exit, implicating the involvement of VSOR channels in BOT‐induced cell volume decrease.

### 3.3 BOT Activates VSOR Channels Endogenously Expressed in HEK293T Cells

**FIGURE 4:**
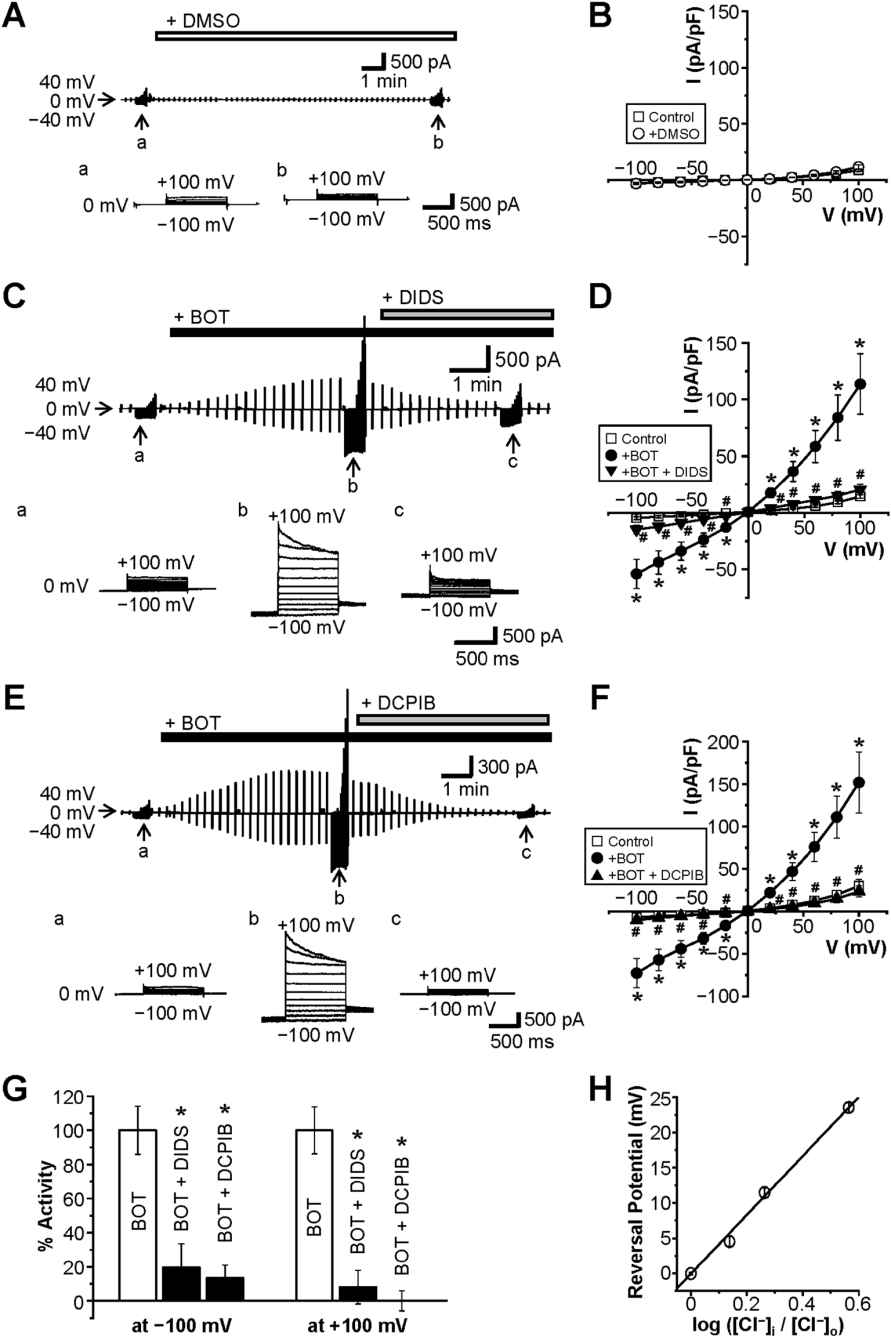
Boi‐ogi‐to (BOT) induces activation of Cl^−^ currents in a manner sensitive to DCPIB and DIDS. Continuous traces of whole‐cell currents in HEK293T cells during the application of alternating pulses from 0 to ±40 mV every 10 s. Traces of current responses to step pulses of 20 mV each from −100 mV to +100 mV at a, b, and c are shown in A, C, and E. (A) Representative recordings in Control cells and those treated with DMSO alone (open bar). (C, E) Representative recordings in the cells treated with BOT (black bars) followed by application of DIDS or DCPIB dissolved in DMSO (gray bars). (B, D, F) Current–voltage (I–V) relationships of mean currents recorded in A, C, and E (*n* = 6–14). (G) Percent currents activated by BOT at −100 mV and +100mV in the absence or presence of DCPIB or DIDS (*n* = 6–14). (H) The reversal potential was measured by varying the extracellular chloride ion concentration ([Cl^−^]_o_) from 110 mM to 80 mM, 60 mM, and 30 mM. The vertical axis represents the reversal potential, while the horizontal axis represents the logarithmic ratio of intracellular to extracellular chloride concentration ([Cl^−^]_i_/[Cl^−^]_o_). Linear fitting analysis indicated that a 10‐fold reduction in [Cl^−^]_o_ led to a + 41.8 mV shift in the reversal potential (*n* = 6–10).

To further investigate the effect of BOT on endogenous Cl^−^ channels, we performed whole‐cell patch clamp experiments in HEK293T cells. BOT administration induced significant activation of Cl^−^ currents that gradually increased over time (Figure 4C,E), whereas DMSO‐treated controls did not show such current activation (Figure 4A,B). The BOT‐induced currents showed a moderately outwardly rectifying current–voltage relationship with a reversal potential at 0 mV under symmetric Cl^−^ concentration conditions (Figure 4D,F). When the extracellular Cl^−^ concentration was reduced from 110 to 80, 60, and 30 mM, the reversal potential of the BOT‐induced currents shifted to the positive potentials with the slope of +41.8 mV/decade (Figure 4H). This shift indicates that Cl^−^ serves as the major charge carrier for these currents.

The BOT‐induced Cl^−^ currents observed upon application of voltage steps (Figure 4Cb,Eb) showed outward rectification and time‐dependent inactivation kinetics at positive potentials larger than +80 mV, a feature consistent with VSOR channels [27]. Furthermore, applications of DIDS and DCPIB effectively blocked BOT‐induced currents (Figure 4C–G), confirming the involvement of VSOR channels.

**FIGURE 5:**
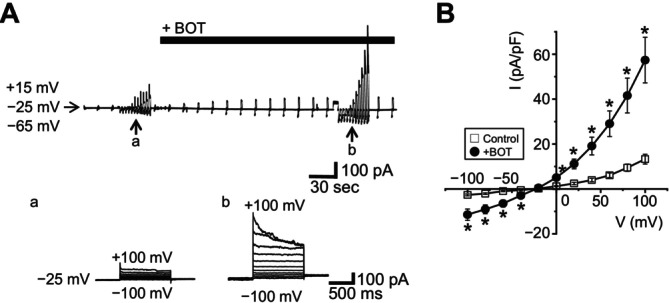
Boi‐ogi‐to (BOT) induces activation of Cl^−^ currents under physiological Cl^−^ conditions. Experiments were performed using a Cl^−^ gradient representative of epithelial cells, with the extracellular chloride concentration ([Cl^−^]_o_) set at 120 mM and the intracellular chloride concentration ([Cl^−^]_i_) at 45 mM. Whole‐cell currents were continuously recorded from HEK293T cells while applying alternating voltage pulses of ±40 mV every 10 s from a holding potential of −25 mV. (A) Representative whole‐cell current traces recorded in cells treated with BOT (black bars). The lower panels (a, b) show current responses to step pulses ranging from −100 mV to +100 mV in 20 mV increments. (B) Current–voltage (I–V) relationships of the averaged currents recorded in (A). The reversal potential was −16.4 ± 0.5 mV (*n* = 7). Statistically significant differences are indicated by *p < 0.05 compared to BOT alone.

We next investigated the BOT‐induced Cl^−^ currents under physiological Cl^−^‐gradient conditions. The intracellular (pipette) Cl^−^ concentration was set at 45 mM, which represents a physiological value observed in epithelial cells, including HEK293 cells [28, 29, 30]. Under this condition as well, BOT activated VSOR Cl^−^ currents (Figure 5) with exhibiting inactivation kinetics (Ab) and outward rectification (B). The reversal potential was −16.4 ± 0.5 mV (*n* = 7) (Figure 5). The resting membrane potential in HEK293T cells, measured using the nystatin‐perforated patch‐clamp technique, was −39.8 ± 0.6 mV (*n* = 8). These results indicate that BOT‐induced activation of inward Cl^−^ VSOR channels occurs at the resting membrane potential, thereby facilitating the efflux of Cl^−^ from the cells.

These electrophysiological properties (e.g., I‐V relationship with outward rectification, inactivation kinetics at large positive voltages, and sensitivity to DIDS and DCPIB) are characteristic of VSOR channels, as summarized in previous review articles [31, 32]. These findings strongly suggest that BOT activates VSOR channels endogenously expressed in HEK293T cells, facilitating Cl^−^ efflux from the cells.

### 3.4 Prerequisite Role of LRRC8A in BOT‐Induced VSOR Activation

**FIGURE 6:**
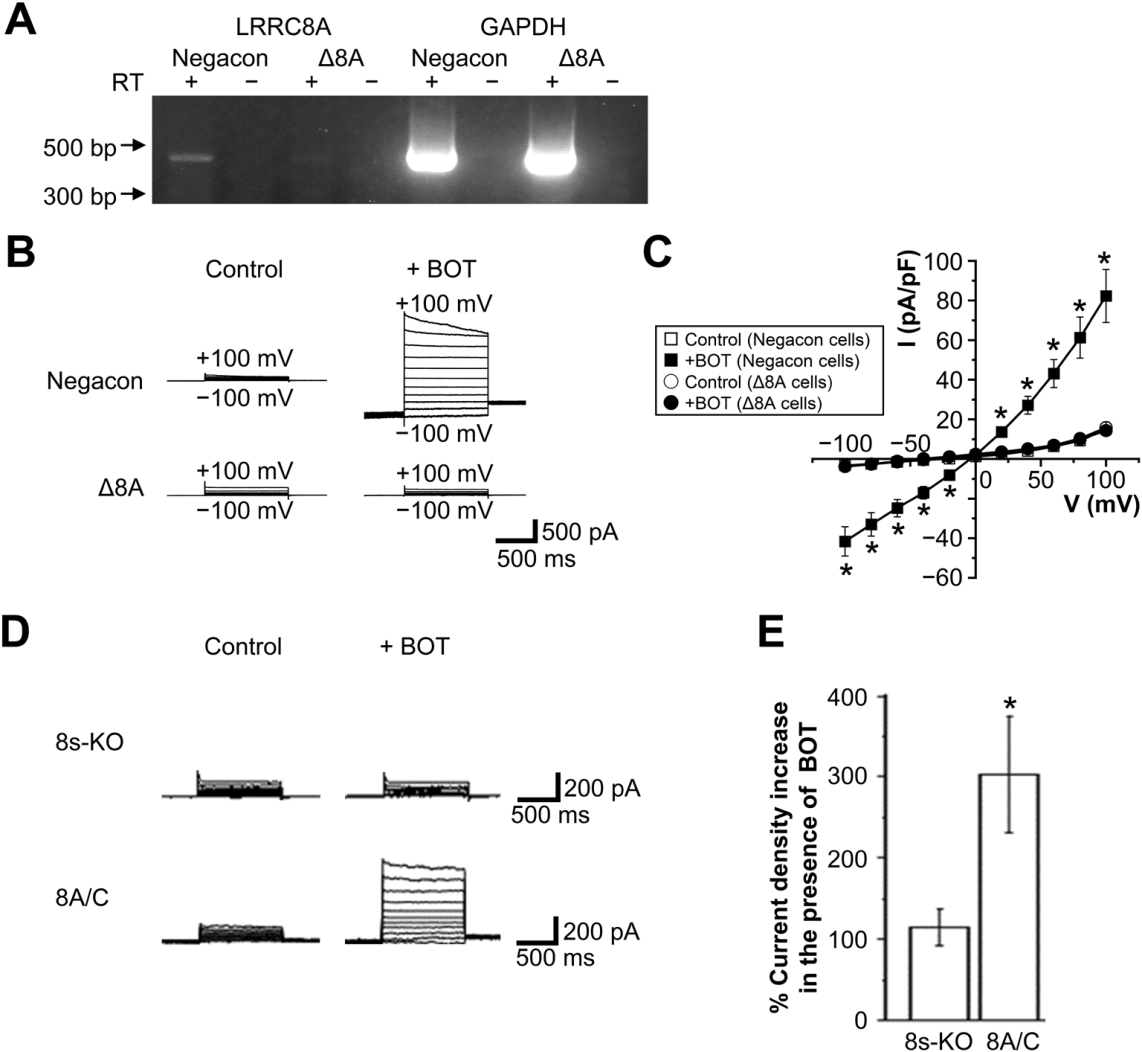
Effects of LRRC8A knockdown and LRRC8A/C overexpression on Boi‐ogi‐to (BOT)‐induced Cl^−^ currents. (A) Representative RT‐PCR results showing expression of LRRC8A mRNA in HEK293T cells transfected with negative‐control siRNA (Negacon) and LRRC8A siRNA (Δ8A). GAPDH is used as a housekeeping gene control. RT(+) and RT(−) represent lanes with and without reverse transcriptase. (B) Representative traces of BOT‐induced whole‐cell currents recorded upon step pulse applications in Negacon and Δ8A cells. (C) Current–voltage relationships for currents recorded in the absence and presence of BOT in Negacon and Δ8A cells (*n* = 8–14). (D) Representative traces of BOT‐evoked whole‐cell currents recorded during step pulse application in LRRC8‐deficient (8s‐KO) cells and in LRRC8A/C‐coexpressing cells. (E) Bar graphs showing peak current densities at +100 mV for the conditions described in (D) (*n* = 6–7). *p < 0.05 compared to Control.

We next explored the effect of LRRC8A knockdown to obtain the molecular evidence for BOT‐induced VSOR activation, since LRRC8A was shown to be a core component of VSOR channels in human cells [12, 33, 34] and mouse cells [35]. HEK293T cells were transfected with siRNA targeting the LRRC8A gene, and the knockdown efficiency was confirmed by a significant reduction in LRRC8A mRNA levels compared to the negative control (Figure 6A: Negacon, ∆8A), consistent with previous studies [14].

As shown in Figure 6B,C, the BOT‐induced Cl^−^ current was significantly suppressed in LRRC8A‐knockdown HEK293T cells (∆8A) compared to control cells, indicating that LRRC8A is essential for BOT‐activated Cl^−^ channels.

To further verify the BOT effect specific on VSOR channels, we performed experiments using LRRC8‐lacking HEK LRRC8^−/−^ cells [13] as a negative control. In these cells, BOT‐induced VSOR currents were not observed as shown in Figure 6 (D, E: 8s‐KO). Since the VSOR activity is known to require the expression not only of LRRC8A but also of LRRC8C, D, and/or E [12] but not of LRRC8B [13, 36], we made positive control experiments by overexpressing both LRRC8A and LRRC8C in HEK LRRC8^−/−^ cells. In contrast to HEK LRRC8^−/−^ cells, LRRC8A/C‐expressing HEK LRRC8^−/−^ cells responded to BOT with Cl^−^ current activation, as shown in Figure 6 (D, E: 8A/C), indicating that the expression of LRRC8 is essential for BOT to activate VSOR currents effectively.

### 3.5 Role of Plasmalemmal Expression of LRRC8A in BOT‐Induced Cell Volume Decrease

**FIGURE 7:**
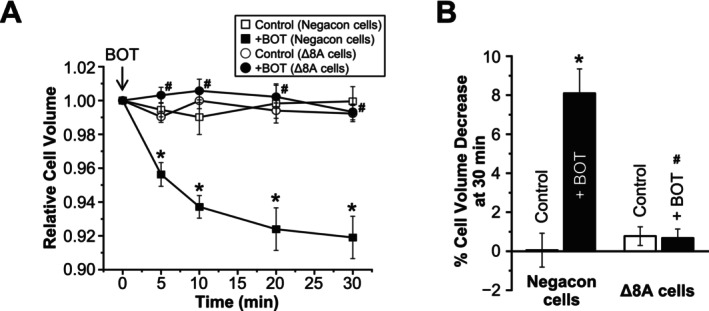
Effects of LRRC8A knockdown on Boi‐ogi‐to‐induced cell volume decrease. (A) Time course of mean cell volume changes in Negacon and Δ8A cells. At time 0 min, BOT (800 μg/mL) was applied except in the control conditions. (B) Percentages of cell volume decrease at 30 min (*n* = 9–17). *p < 0.05 compared to Control in Negacon cells; ^#^ *p* < 0.05 compared to BOT‐treated Negacon.

Then, to assess the role of LRRC8A in BOT‐induced cell volume decrease, we performed cell volume measurements in LRRC8A‐knockdown cells. As shown in Figure 7, BOT‐induced cell volume decrease was abolished entirely in cells treated with siRNA against the LRRC8A gene, confirming the crucial role of LRRC8A in this process.

**FIGURE 8:**
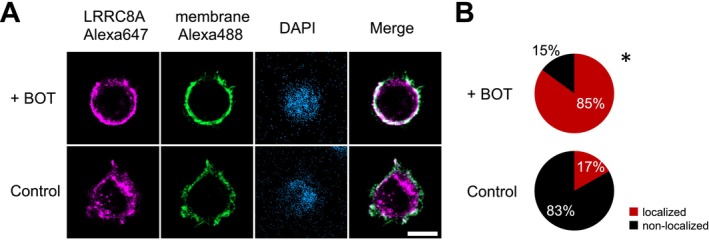
Effects of Boi‐ogi‐to (BOT) on the expression pattern of LRRC8A protein within HEK293T cells. (A) Confocal images of HEK293T cells stained with anti‐mCherry antibody (hLRRC8A‐mCherry: Red) and anti‐GFP antibody (EGFP‐F: Green) in the presence or absence of BOT (800 μg/mL). Nuclei are stained with DAPI (blue). Merge shows the overlay of LRRC8A‐mCherry and EGFP‐F images. Scale bar: 10 μm. (B) BOT treatment increases the localization of LRRC8A to the cell periphery region corresponding to the plasma membrane compared to cells without BOT treatment. Statistical significance was determined by chi‐square test (*p < 0.001). A total of 67–237 cells were observed and analyzed.

Finally, we assessed the membrane localization of LRRC8A protein in HEK293T cells transfected with LRRC8A‐mCherry. In the absence of BOT, LRRC8A was predominantly localized in the intracellular space, but upon BOT treatment, LRRC8A became significantly localized to the cell periphery region, including the plasma membrane after 10 min (Figure 8A). The merged images further confirmed that LRRC8A stained with Alexa647 colocalized with the plasma membrane stained with Alexa488 after the application of BOT, supporting the inference that BOT enhances plasmalemmal localization of LRRC8A (Figure 8B).

These results clearly demonstrate that BOT induces plasmalemmal expression of LRRC8A and thereby functions as an activator of VSOR channels, with LRRC8A being a key component in mediating BOT‐induced cell volume decrease.

## 4 Discussion

In this study, we demonstrated for the first time that BOT, a traditional Japanese herbal medicine, promotes Cl^−^ efflux from the epithelial cells by activating VSOR channels, also known as VRACs. Our findings indicate that BOT‐induced cell volume decrease occurs in a dose‐dependent manner (Figure 1D), primarily through the activation of VSOR channels, which was confirmed by the sensitivity of the Cl^−^ current to VSOR inhibitors such as DIDS and DCPIB (Figure 4). Moreover, knockdown of the LRRC8A gene, a core component of VSOR channels, significantly attenuated BOT‐induced Cl^−^ currents (Figure 6A–C) and cell volume decrease (Figure 7), and we reproduced BOT‐induced currents in LRRC8‐deficient cells expressing LRRC8A/C (Figure 6D,E). These results further solidify the involvement of VSOR channels in the action of BOT.

To our knowledge, this is the first report demonstrating the activation of anion channels by BOT and the first indication that BOT targets VSOR channels. VSOR channels play a key role in maintaining cell volume homeostasis by mediating Cl^−^ transport in various tissues [32, 33]. The present new finding is that BOT can activate VSOR channels without cell swelling but even with cell shrinkage (Figure 1). This fact adds a novel mechanism of action to the pharmacological profile and therapeutic action of this traditional medicine.

VSOR channels are heteromeric complexes composed of LRRC8 protein members, with LRRC8A being essential for channel function [12]. While the molecular identity of these channels has been found only recently, their physiological relevance has been extensively studied. VSOR channels are activated under conditions of cell swelling to prevent excessive cellular expansion and to restore normal cell volume, called the regulatory volume decrease (RVD), or apoptotic conditions exhibiting continuing cell shrinkage, called apoptotic volume decrease (AVD), by facilitating Cl^−^ and organic anion effluxes [32]. However, BOT‐induced activation of VSOR and persistent cell shrinkage never resulted in apoptotic cell death (Figure 2).

Our findings, thus, suggest that the mechanism of VSOR activation by BOT is distinct from these conventional stimuli. Specifically, we demonstrated that BOT induces the translocation of LRRC8A to the plasma membrane (Figure 8) and activates VSOR channels without osmotic or apoptotic stimulation. This novel mechanism of VSOR channel activation by BOT expands our understanding of the ways in which traditional medicines can influence cellular physiology, particularly in terms of ion transport and cell volume regulation.

One may deem a possibility of BOT‐induced activation of other Cl^−^ channel types, such as the acid‐sensitive outwardly rectifying Cl^−^ channel (ASOR), Ca^2+^‐activated Cl^−^ channel (CaCC), cAMP‐activated anion channel CFTR, and large‐conductance Maxi‐Cl channel. However, the mild outward rectification, which is the most characteristic biophysical property of VSOR, observed here upon BOT stimulation is distinct from the sharp outward‐rectifier ASOR and CaCC, as well as from the ohmic (I‐V linear) CFTR and Maxi‐Cl [37]. Time‐dependent inactivation at large positive potentials observed with BOT is consistent with VSOR currents but inconsistent with ASOR and CaCC, which exhibit activation, not inactivation, kinetics at positive potentials and with CFTR, which does not exhibit time‐ and voltage‐dependent changes [37]. Since no BOT‐induced currents were detected in LRRC8‐deficient HEK cells, above all, we conclude that the involvement of other anion channels can be clearly ruled out. In addition, because the BOT‐induced current could be reproduced in HEK LRRC8^−/−^ cells expressing LRRC8A/C, it appears that the BOT‐induced current can activate LRRC8A/C‐containing VSOR.

Notably, our cell viability assays demonstrated that BOT‐induced cell volume decrease occurred without triggering cell death (Figure 2A,B). Interestingly, BOT‐treated cells even showed intact cell proliferation (Figure 2C), suggesting that BOT may not only be safe but also beneficial for cell survival and growth. This observation reinforces the clinical safety profile of BOT and hints at the possibility of broader therapeutic applications, where BOT could promote fluid excretion without compromising cell viability and proliferation.

It is well known that the ability of proper cell volume regulation is essential for cell viability and proliferation [38, 39]. Additionally, BOT contains bioactive compounds, such as flavonoids, which may support cell proliferation by activating intracellular signaling pathways, including growth factors. In other types of Kampo medicines, naringin and genistein, which promote the proliferation of human bone marrow mesenchymal stem cells, are known to promote the proliferation of bone marrow‐derived stem cells [40], and these components are found in the citrus herbal medicines orange peel, citrus fructus fruit, and cherry bark herbal medicine, cherry bark, respectively.

Given the essential role of epithelial and vascular endothelial cells in regulating fluid balance, enhanced proliferation of these cell types by components contained in BOT could contribute to vascular integrity, tissue repair, and improved microcirculation, thereby accelerating edema resolution. Further research is needed to explore whether BOT‐induced cell proliferation plays a direct role in its anti‐edema effects.

The fact that BOT activates VSOR channels also opens the door to potential therapeutic applications beyond water excretion. VSOR channels are expressed in tissues such as the kidney, heart, brain, and blood cells, and play essential roles in physiological processes including volume regulation, organic compound excretion, and apoptosis induction [15, 41, 42, 43, 44, 45, 46, 47]. Dysregulation of the VSOR channel function has been implicated in a range of pathological conditions, including cancer progression, drug resistance, neurological disorders, and myocardial remodeling [48, 49, 50, 51]. Given its ability to activate VSOR, BOT could be explored as a treatment for conditions associated with fluid retention, such as edema and nephrotic syndrome, as well as diseases, such as certain cardiovascular and neurological conditions, where VSOR may play a protective role.

BOT does not promote Cl^−^ secretion from all cell types. Instead, it triggers water secretion in intestinal and renal epithelial cells such as human colonic Caco‐2 and mouse renal tubular cells, but not in human cervical cancer HeLa cells (see Figure S1). This points to its action on VSOR channels expressing in mesothelial cells that represent the epithelial cells covering the body cavity like the renal tubules and digestive tract. This selective action may be attributed to the recent finding that LRRC8A, a core component of VSOR channels, is predominantly expressed in proximal tubules [41].

The determination of which is the BOT role in Cl^−^ excretion or (re)absorption within actual tissues, such as the kidney and intestine, remains challenging due to their intrinsic epithelial polarity. Nonetheless, our findings suggest that BOT may promote Cl^−^ (re)absorption in these tissues, in light of the clinical data demonstrating increased plasma Cl concentrations following BOT administration (see Tables 2 and 3).

Specifically, BOT appears to enhance Cl^−^ reabsorption in the kidney, thereby increasing plasma Cl^−^ levels and subsequently elevating plasma NaCl concentrations. This physiological change contributes to increased intravascular osmolality, which facilitates the movement of excess interstitial water into the circulation. Such a classic mechanism [52] may explain the anti‐edema effects observed in association with BOT. Notably, this effect occurs independently of changes in albumin concentration, which is the primary determinant of oncotic pressure (Table 2).

Our study further demonstrated that BOT administration increased plasma NaCl levels within 4–5 weeks (see Tables 2 and 3), coinciding with the tendency of a rise in blood pressure during prolonged treatment (Table 2). These BOT effects observed within 4–5 weeks align with previous clinical evidence, including a study on gynecological cancer patients with deep vein thrombosis and post‐thrombotic syndrome, where BOT was shown to be effective in alleviating lower limb edema within 4–6 weeks [19].

Although the changes in plasma Na and Cl concentrations appear relatively transient and recover after 6–8 weeks, the cellular‐level effects of BOT may persist, as its therapeutic benefits were reported to remain evident after 6 weeks in humans in vivo [19]. However, prolonged ion mobilization from the interstitial fluid into the bloodstream may contribute to an increase in blood pressure over time. This could be attributed to remodeling of small resistance arteries and structural and functional changes in large elastic arteries due to plasma volume expansion through renin‐angiotensin system activation [53, 54]. Therefore, additional clinical trials are required to determine whether long‐term BOT administration is necessary for sustained efficacy while ensuring safety.

Consequently, the anti‐edema effects of BOT may involve selective ion regulation within epithelial cells, thereby reducing tissue swelling through the modulation of vascular osmolality. While it is essential to consider other mechanisms contributing to peripheral swelling, such as alterations in vascular permeability [55], future experimental evidence could substantiate this ion transport‐based hypothesis, particularly through studies utilizing polarized renal and intestinal epithelial cells.

In conclusion, our study may provide valuable insights into the molecular mechanisms by which BOT exerts its therapeutic effects. By activating VSOR channels, BOT promotes Cl^−^ efflux and reduces cell volume, likely contributing to its clinical efficacy in promoting water excretion. These findings not only enhance our understanding of the pharmacological actions of BOT but also highlight the broader potential for VSOR channel activation as a therapeutic strategy in various diseases. Future research should focus on elucidating the detailed molecular mechanisms of VSOR activation by BOT and exploring its therapeutic potential in a broader range of clinical applications.

## Author Contributions

K.S.‐N. conducted all experiments and analysis. T.S., H.S., S.K., and A.S. contributed to the investigation, writing, reviewing, editing, and validation of the findings. S.M., H.N., H.H., H.S., and Y.O. helped to design the work and commented on the draft. T.N. conceived and designed the work and wrote the manuscript.

## Disclosure

Declaration of Transparency and Scientific Rigor: This declaration acknowledges that this paper adheres to the principles for transparent reporting and scientific rigor of preclinical research recommended by funding agencies, publishers, and other organizations engaged with supporting research.

## Conflicts of Interest

The authors declare no conflicts of interest.

## Supporting information


**Figure S1.** Effects of Boi‐ogi‐to (BOT) on cell volume decrease in different cell types and the impact of administration of Cl^−^ channel inhibitors.

## Data Availability

The authors have nothing to report.
