# Supplementary material for: Boi‐Ogi‐To, a Traditional Japanese Kampo Medicine, Promotes Cellular Excretion of Chloride and Water by Activating Volume‐Sensitive Outwardly Rectifying Anion Channels
Source: FASEB J. 2025 May 8;39(9):e70573. doi: 10.1096/fj.202403278R (PMC12059615; doi:10.1096/fj.202403278R)
Supplement: Supplementary file 1 — Figure S1. Effects of Boi‐ogi‐to (BOT) on cell volume decrease in different cell types and the impact of administration of Cl− channel inhibitors. [file FSB2-39-e70573-s001.docx]

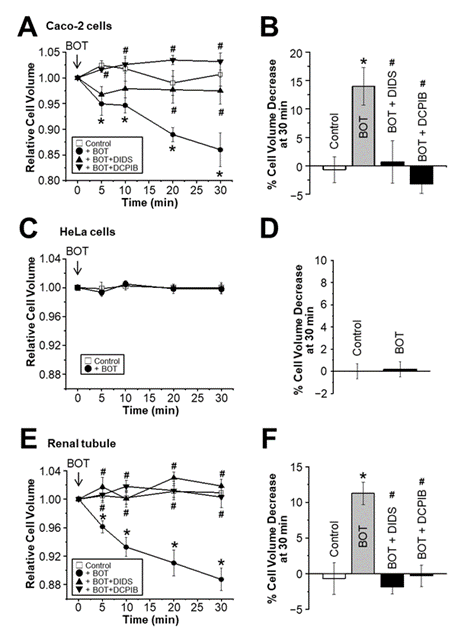


**Supplementary Figure 1**

**Effects of Boi-ogi-to (BOT) on cell volume decrease in different cell types and the impact of administration of Cl**^−^ **channel inhibitors.** (Left panels) Time courses of cell size changes in Caco-2 cells (A) and of mean cell volume changes in HeLa cells (C), and of the mean cross sectional area (CSA) in mouse renal tubular epithelial cells (E). BOT (800 μg/ml) was applied at 0 min, with 100 μM DIDS or 5 μM DCPIB together with BOT, except in the control condition. (B, D, F) Percentages of cell volume changes (B, D) or CSA changes (F) measured at 30 min after BOT administration, calculated from the data in A, C, and E, respectively (n=7-12). *P < 0.05 compared to Control; ^#^P < 0.05 compared to BOT alone.
